# Novel concept of personalized therapy with continuous positive airway pressure

**DOI:** 10.1007/s11325-024-03095-0

**Published:** 2024-07-01

**Authors:** Motoo Yamauchi, Yasutaka Moritsuchi, Eriko Hamada

**Affiliations:** 1 Department of Clinical Pathophysiology of Nursing, Nara Medical University, 840 Shijocho, Kashihara, Nara, 634-8521 Japan; 2 Department of Respiratory Medicine, Nara Medical University Hospital, Nara, Japan; 3 Sleep Center, Kirigaoka Tsuda Hospital, Kitakyushu, Japan

**Keywords:** CPAP, CPAP adherence, Personalize therapy

Continuous positive airway pressure (CPAP) has been established as a standard therapy for moderate to severe obstructive sleep apnea (OSA). CPAP improves obstructive apneic events with favorable effects on co-morbidities including cardiovascular diseases, arrhythmias, metabolic and cognitive dysfunction, as well as mortality. However, the problem is suboptimal CPAP adherence. Only 17 to 54% of patients with OSA have been reported to adhere to CPAP when adherence is defined as at least 4 h of use per night [1]. This issue inspired many physician-scientists to explore the strategy to improve CPAP adherence. In the meantime, the recognition of existing various OSA phenotypes has spread widely. Thus, personalized therapy according to the identified phenotype has been focused. Furthermore, given the suboptimal adherence of CPAP, other therapeutic approaches as an alternative therapy of CPAP have been hot topics for personalized therapy. A growing body of studies using pharmacological intervention and device-based therapy such as hypoglossal nerve stimulation have been reported to improve OSA. However, as we will propose the novel concept of “Personalized Therapy with CPAP” in this editorial, there are still many things to do sticking with CPAP before stepping forward to alternative therapies of CPAP.

Previous studies have demonstrated strategies to improve CPAP adherence. Earlier intervention of two to four weeks after CPAP implementation improved long-term CPAP adherence [2, 3]. Others reported that cognitive behavioral therapy, telemonitoring, and patient engagement technology and programs improved CPAP adherence [4–6]. However, none of those studies considered specific properties of used CPAP. Features of the flow generator and pressure change algorithm of auto-titrating CPAP (APAP) differ between CPAP manufacturing companies [7, 8]. In daily clinical practice, we sometimes experience that replacing the CPAP machine with other companies’ machines improves CPAP adherence, implying that a preference for a CPAP machine might differ among OSA patients and phenotypes.

Currently, most of the prescribed CPAP machine is APAP machine with either fixed or auto mode. In the case of patients who are prescribed APAP with fixed mode, the specific feature of the flow generator affects breathing comfortability, because positive pressure inside the mask varies widely during inhalation and exhalation depending on how much tidal volume the patient inhales and respiratory rate as well. The flow generator automatically adjusts the output by raising and lowering rpm over time during inhalation and exhalation to maintain the set positive pressure level inside the mask. Breathing comfortability would be affected by how quickly the flow generator responds supplementing the pressure during inhalation. Thus, a sleep physician would need to assess not only the tidal volume or body weight which can estimate tidal volume but also the respiratory rate during sleep by re-reviewing the diagnostic polysomnography (PSG). For most patients, an APAP machine with auto-mode is used. In this case, in addition to the feature of the flow generator, pressure change algorithms would affect comfortability with CPAP during sleep. Recently, a new CPAP machine named Murata MX (Murata Manufacturing Co. Ltd. Kyoto, Japan) has become available in Japan. Murata MX equips three automatic pressure change algorithms, which are soft, normal, and hard modes. Physicians can choose and change the three algorithms. A bench test study using RespiSim-System ASL5000 (IngMar Medical, Pittsburgh, Pennsylvania) was performed to see the responses according to simulated sleep-disordered events using several APAP devices with auto mode. The pressure range was set to 4–20 cmH_2_O. Regarding the pressure response by simulated obstructive apnea, Fig. 1 shows a substantial difference between DreamStation (Philips Respironics, Murrysville, Pennsylvania) and AirSense 10 (ResMed, San Diego, California). Also, the hard mode of Murata MX behaves like AirSense 10 in terms of pressure increase rate, but the difference is that Murata MX has an apnea cap at the point of 14cmH_2_O. The pressure increase rate of DreamStation is similar to the Murata MX of soft mode. Furthermore, Murata MX with a normal mode increases the pressure with the intermediate rate between DreamStation and AirSense 10. Pressure responses according to the hypopnea with flow limitation are shown in Fig. 2. It is likely seen the substantial difference between DreamStation and AirSense 10, and the pressure response rate of Murata MX with a hard mode is close to that of AirSense 10. The pressure response rate tends to decline gradually with the modes of normal and soft.

**Fig. 1:**
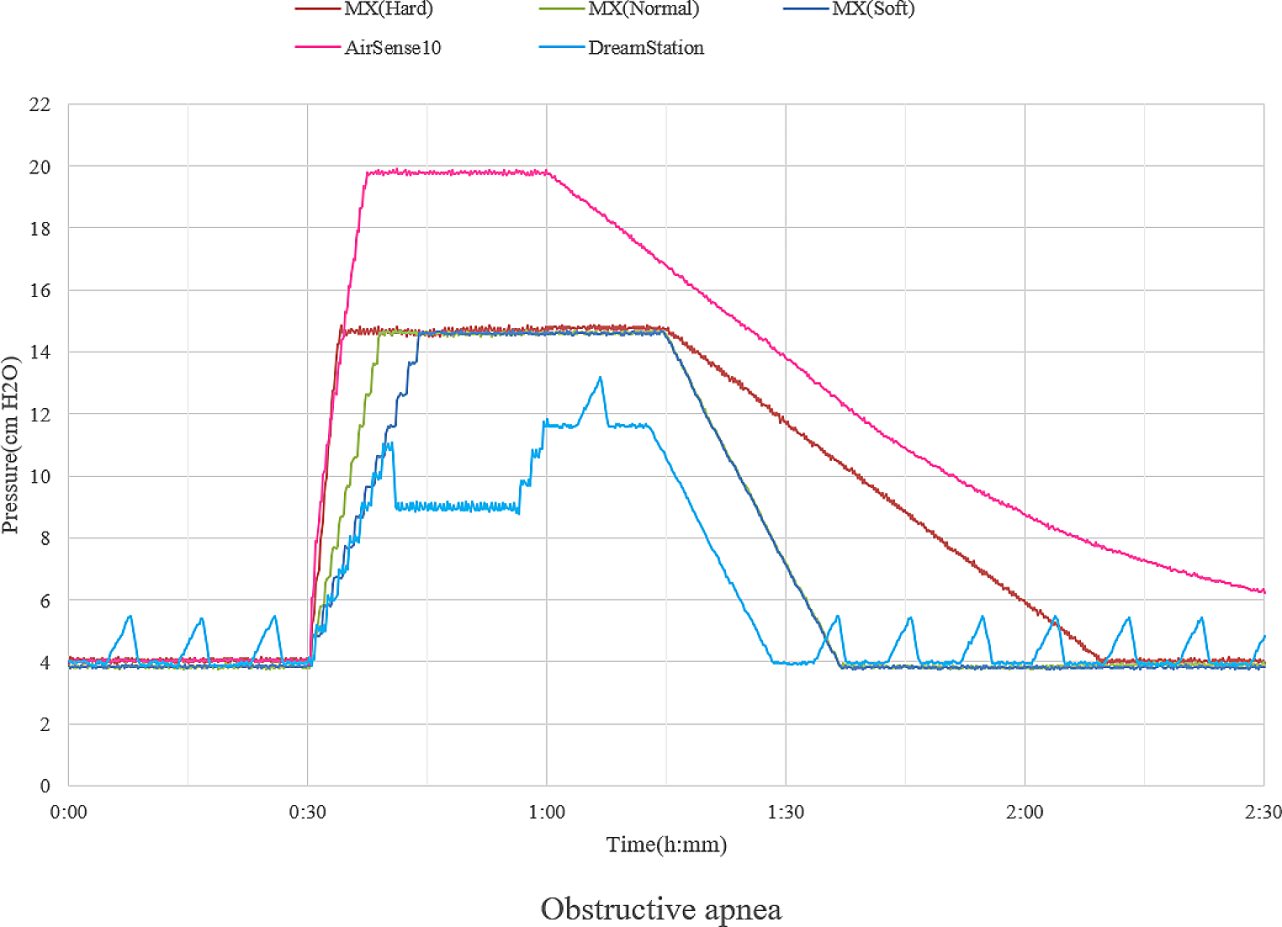
APAP pressure response according to the simulated obstructive apnea. Every APAP device setting is 4-20 cmH_2_O. The color bar represents APAP machines (ResMed and Philips) and three algorithms for pressure change of Murata MX. Puressure increase rate of Murata MX with hard mode and soft mode is similar to the one of AirSense 10 and DreamStation, respectively. APAP; auto-titrating positive airway pressure

**Fig. 2:**
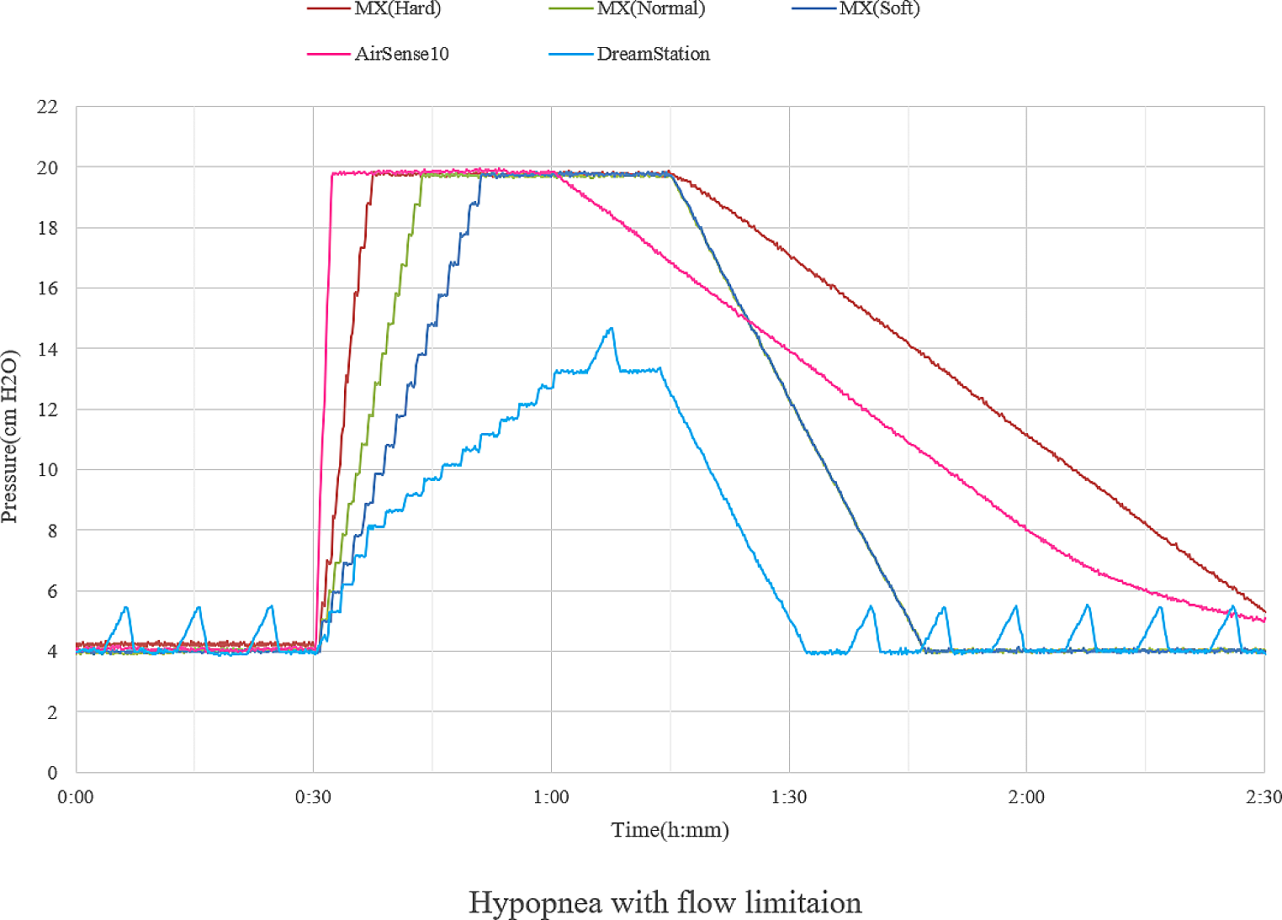
APAP pressure response according to the simulated hypopnea with flow limitation. Every APAP device setting is 4-20 cmH_2_O. The color bar represents APAP machines (ResMed and Philips) and three algorithms for pressure change of Murata MX. The pressure increase rate is steeper with Airsense 10, but close to the hard mode of Murata MX. Then, the order of pressure increase rate is declining with the normal mode and soft mode of Murata MX, and DreamStatuion. APAP; auto-titrating positive airway pressure

The CPAP adherence would differ between OSA phenotypes. For instance, a rapid increase in providing pressure of APAP might interrupt sleep continuity for a patient whose endotype is a low arousal threshold. Conversely, a slow pressure increase rate might be insufficient to restore an apneic event, especially in patients with morbid severe OSA with severe upper airway collapsibility and insufficient upper airway compensation. Accordingly, we believe we should go back to staring at the suitability of each patient with a given CPAP device or CPAP mode before we consider alternative therapy of CPAP. The proposal of this editorial is “Personalized Therapy with CPAP” at the point of the earlier step of “Personalized Therapy of OSA”. Considering these issues, sleep physicians may want to prescribe the APAP device which can modify multiple algorithms of pressure increase rate without replacing the APAP device with another company’s machine. However, further study should be needed to elucidate which algorithm is suitable for a specific OSA phenotype to establish “Personalized Therapy with CPAP”.

## Electronic supplementary material

Below is the link to the electronic supplementary material.

Supplementary Material 1
